# 

**Published:** 1843-01

**Authors:** 


### CONTENTS

OF NO. VII.

### ORIGINAL COMMUNICATIONS.

ESSAYS AND CASES.

Art. I.—An Experimental and Critical Inquiry into the Nature and Treatment of wounds of the Intestines. By Samuel D. Gross, M. D., Professor of Surgery in the Louisville Medical Institute. - - - 1

SELECTIONS FROM AMERICAN AND FOREIGN JOURNALS.

Virey's Objections to Liebig's Theory of the Uses of Respiration and of the food. - - - - - 51
Phloridine. - - - - - - 52
Treatment of the Hemorrhagic Diathesis. - - - 53
Deafness cured by the endermic use of Morphia. - - 54
On Percussion. - - - - - - 54
New Remedy for Scalds and Burns. - - - 58
Analysis of the Menstrual Fluid. - - - - 59
Revivification of Microscopic Animalculæ. - - 59
Corpora Lutea. - - - - - - 60
Signs of Maturity in new-born Children. - - - 61
On a peculiar affection of the Cornea in Nurses. - - 61

ORIGINAL INTELLIGENCE.

Western Meteorology. - - - - - - 63
Temperature and Diseases of the year 1842. - - - 64
Pneumonia of the Winter of 1841–'2. - - - 65
Northern limits of Milk-sickness. - - - - - 65
Snake-bites. . - - - - - 65
Throat Disease of Public Speakers. - - - 66
Abolition of a rule in Medical Graduation.—Willoughby University of Lake Erie. - - - - 68
Saturated Solution of Corrosive Sublimate in Erysipelas. - 72
A Discovery of the true cause of the Disease called by the people Trembles or Milk-sickness. - - - 73
School for the Blind. - - - - - 77
The Mammoth Cave a winter resort for Invalids. - - 78
Lithotomy on a Man seventy-eight years of age. - - 78
Progress of Temperance. - - - - - 79
To Professional Correspondents. - - - - 79
Miscellaneous Notices. - - - - - 80

